# Sex‐Biased Interactions Between Spur‐Thighed Tortoises (*Testudo graeca*) and Hindgut Nematodes

**DOI:** 10.1002/ece3.74377

**Published:** 2026-09-28

**Authors:** Julieta Benítez‐Malvido, Eva Graciá, Andrés Giménez, Iraís Ávila‐Eulogio, Diana María Méndez, Roberto Carlos Rodríguez‐Caro, Rocío Ruiz‐De Ybáñez, Anna Traveset

**Affiliations:** ^1^ Laboratorio de Ecología del Hábitat Alterado, Instituto de Investigaciones en Ecosistemas y Sustentabilidad Universidad Nacional Autónoma de México Morelia Michoacán Mexico; ^2^ Departamento de Biología Aplicada, Facultad de Ciencias Experimentales Universidad Miguel Hernández Elche Alicante Spain; ^3^ Centro de Investigación e Innovación Agroalimentaria y Agroambiental (CIAGRO‐UMH) Universidad Miguel Hernández Elche Alicante Spain; ^4^ Departamento de Sanidad Animal, Facultad de Veterinaria, Campus de Excelencia Internacional Regional “Campus Mare Nostrum” Universidad de Murcía Murcía Spain; ^5^ Global Change Research Group Institut Mediterrani d'Estudis Avançats (CSIC‐UIB) Esporles, Mallorca Balearic Islands Spain

**Keywords:** biotic interactions, hindgut nematode community, host‐nematode networks, Mediterranean landscapes, sexual dimorphism

## Abstract

The nature of endosymbiont interactions is driven by different ecological and evolutionary processes. In herbivorous reptiles exhibiting sexual dimorphism, it remains unclear whether the host's sex and habitat type influence their interactions with hindgut nematodes. To explore this, we sampled spur‐thighed tortoises ( *Testudo graeca* ) across different land‐use practices in southern Spain (Mediterranean shrublands, traditional and intensified agricultural fields, pine plantations, and unproductive lands). For male and female tortoises, we assessed the relationships between nematode infestation and body traits (weight, height, plastron width, and carapace length) and growth rates across different land uses. Further, we investigated differences in species composition and diversity of hindgut nematode communities between sexes. Finally, with an intrapopulation ecological network approach, we evaluated the structure and diversity of tortoise‐nematode interactions by focusing on nematode species infesting individual male and female tortoises. The results revealed a sex‐mediated relationship between nematodes and tortoises. Although nematode prevalence and infestation were similar between sexes, infestation was negatively related to body size and weight only in males. At low levels of nematode infestation, males grew faster than females; however, as infestation increased, male growth rates declined, whereas female growth remained stable. In both sexes, nematode infestation decreased with the proportion of unproductive lands, and growth rates increased significantly in response to this landscape characteristic. Females hosted significantly more diverse and evenly distributed nematode communities than males despite low compositional dissimilarity between sexes. Nestedness was greater in female‐nematode networks, suggesting that nematodes can be more easily transmitted across these networks compared with males. Overall, females seem more resilient to nematode infestation than males. These findings have important implications for understanding the complex interactions between hindgut nematodes, habitat loss and host behavioral and ecophysiological traits, which emphasize the need for sex‐directed conservation approaches for tortoises.

## Graphical abstract

Tortoise ( *Testudo graeca* ) responses to nematode infestation are clearly sex‐biased, where females seem more resilient to nematode infestation than males. These findings emphasizes the need for sex‐directed conservation approaches in tortoises.

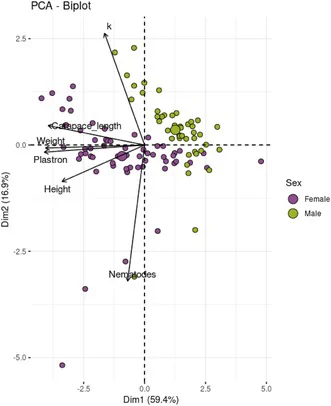

## 1 Introduction

Biotic interactions drive many ecological and evolutionary processes in natural ecosystems and are vitally important for maintaining biodiversity as they affect several population and community attributes over a wide range of spatiotemporal scales (Dallas et al. 2018; Fischhoff et al. 2020; Laine and Tylianakis 2024). Broadly, biotic interactions can be grouped into three types: mutualistic, antagonistic, and commensal. In particular cases, the same interacting animal species can be involved in one or more interaction types (Benítez‐Malvido et al. 2019; O'Grady et al. 2005; O'Grady and Dearing 2006). For example, the same nematode species (*Parapharyngodon riojensis*) could have commensalistic or mutualistic interactions with Liolaemid lizards (O'Grady and Dearing 2006); whereas some bacteria (*Salmonella* spp.) could be commensal as well as pathogenic in some tortoise species (Jacobson 1994). In some herbivorous reptiles such as chelonians, interactions with hindgut oxyurid nematodes (endosymbionts) have been categorized as commensal, mutualistic, and/or pathogenic (O'Grady et al. 2005; Benítez‐Malvido et al. 2019). Only for captive tortoises has hindgut nematode infection been found to be pathogenic and life threatening (Chávarri et al. 2012; Jacobson 2007; Loukopoulos et al. 2007; Rideout et al. 1987). In the Iberian Peninsula, the interaction type of the spur‐thighed tortoise ( *Testudo graeca* Linnaeus, 1758) with oxyurid nematodes has been shown to depend on the level of habitat loss and fragmentation, which affects growth rates (Rodríguez‐Caro et al. 2013, 2017; Benítez‐Malvido et al. 2019). Specifically, nematode infestation was linked to increased tortoise growth in preserved habitats, suggesting a mutualistic relationship. In disturbed habitats, however, this relationship became negative. Furthermore, at low levels of infestation, tortoises grew faster in disturbed habitats than in preserved ones (Benítez‐Malvido et al. 2019). These findings, however, did not account for differences between males and females.

Antagonistic interactions, such as those between parasites and a vertebrate host species, depend on behavioral, physiological and ecological factors (Collinge et al. 2005; Dallas and Presley 2014; Dallas et al. 2018; Fischhoff et al. 2020). In a wide range of vertebrate species, differences in parasite prevalence (i.e., percentage of infected hosts), intensity (i.e., mean number of parasites per infected host), and the effect on host health are often sex‐biased (Rosso et al. 2020). In wild animal populations such as birds and mammals, sexually mature males are generally more susceptible to infection and carry higher parasite loads than females (Poulin 1996; Gosnell et al. 2009; Zuk 2009). This increased susceptibility is partly due to male's physiology, since there is a negative association between testosterone levels and the immune system. Also, males exhibit increased stress when displaying for mating (Declerck et al. 2014; Forbes 2020; Rosso et al. 2020; Zuk 2009). Regarding host behavior and ecology, males and females differ in their breeding and foraging behavior which may influence their abundance, distribution, diet and sociability (Ezenwa and Jolles 2011; Gosnell et al. 2009; Zuk and McKean 1996; Zuk 2009). Thus, sex‐driven behavior and ecophysiological traits could influence parasite ecology, exposure and transmission (Rosso et al. 2020).

In the highly transformed Mediterranean landscapes of southern Spain, wild populations of *T. graeca* tortoises show different sexual traits, including sexual dimorphism, diet, and movement behavior (Anadón et al. 2012; Roca 1999; Semaha et al. 2024). Females are larger and heavier than males and often have more specific nutrient needs for egg development (Gosnell et al. 2009). Conversely, males cover larger distances for mating, whereas females move within patches that provide greater food quality and places for egg laying (Anadón et al. 2006). Nevertheless, both males and females are sensitive to habitat loss and fragmentation (Anadón et al. 2006; Benítez‐Malvido et al. 2019). In this study, we used free‐ranging, naturally infested adult individuals of spur‐thighed tortoises and their hindgut nematodes as a research model to determine if the interaction type between nematode infestation, tortoise body traits and growth rates is influenced by host sex and habitat type.

Given the high prevalence of hindgut nematodes and the presence of sexual dimorphism in wild *T. graeca* populations, we investigated the tortoise‐nematode interaction by considering nematode community attributes and host‐nematodes ecological interaction networks (Benítez‐Malvido et al. 2019). The aims of the study were the following: (1) to identify the interaction type between tortoises and nematodes (i.e., mutualistic, commensal and/or antagonistic) in relation to tortoises´ body traits, growth rates, sex and land‐use practice; (2) to describe the nematode community attributes infecting male and female tortoises (i.e., the pathobiome, *sensu* Vayssier‐Taussat et al. 2014; Chao et al. 2020); and (3) to determine if the topology of host‐nematode ecological networks is driven by tortoise sex.

Because of their different behavioral and ecophysiological traits, we expected that growth rates, nematode communities, and host‐nematode interactions would vary according to sex and land‐use practice. There are very few published epidemiological studies on nematode infestation in wild *T. graeca* populations, where transmission dynamics and impact to their hosts may differ substantially from those observed in captivity (Chávarri et al. 2012; Gómez and Nichols 2013). Furthermore, the relevance of sex has not been a considered factor in driving the type of host‐nematode interactions in tortoises. This study provides a multifaceted approach as we integrated host traits, landscape context, nematode community composition, and ecological network structure in male and female individuals.

## 2 Materials and Methods

### 2.1 Study Area

The study region for the *T. graeca* southeastern Spanish population comprises approximately 2600 km^2^ of semiarid coastal mountains between the Almeria and Murcia provinces (longitude N38° 23 to N36° 20′ and latitude W0° 30 to W2° 20′, Figure 1). The climate is semiarid with mild winters, hot summers and low rainfall (200–350 mm/year) during the cooler months. This region has endured different human interventions for centuries, but it has remained a biodiversity and speciation hot spot (Blondel 2006; Hewitt 2011; Myers et al. 2000). Several plant and animal species are currently threatened with extinction because of the increased conversion of Mediterranean shrublands to agricultural and other land uses (Anadón et al. 2006; Rodríguez‐Caro et al. 2017). Intensified agricultural practices, fires and reforestation with pines have affected the growth and survival of *T. graeca* populations (Anadón et al. 2006; Rodríguez‐Caro et al. 2013, 2017; Sanz‐Aguilar et al. 2011). Nevertheless, some of these anthropogenic landscapes may provide habitat and refugia for several regional endangered species (Agnoletti and Santoro 2022).

**FIGURE 1:**
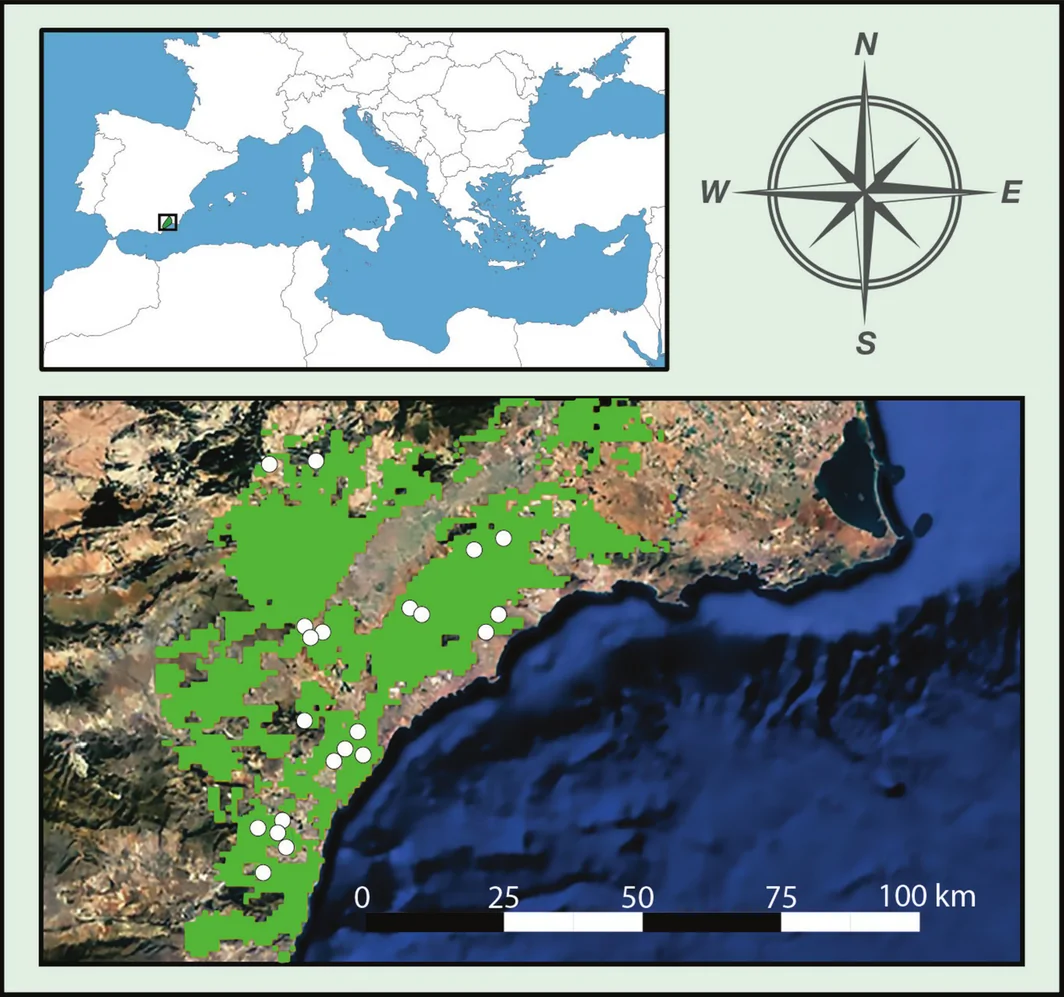
Map showing the distribution of the spur‐thighed tortoise ( *Testudo graeca* ) in the Mediterranean basin. White dots indicate the wild populations localities. Such populations cover approximately 2600 km^2^ of semiarid coastal mountains between the Almeria and Murcia provinces.

### 2.2 The Spur‐Thighed Tortoises and the Prevalence of Nematodes

The spur‐thighed tortoise ( *T. graeca* ) is one of the most widely distributed species of terrestrial tortoises (Graciá et al. 2017). This medium‐sized tortoise of approximately 25 cm in length typically inhabits arid and semiarid shrublands. It is severely threatened throughout its entire distribution range because of habitat loss, fragmentation, and land‐use changes, as well as illegal collection (Anadón et al. 2006; Anadón et al. 2012; Lovich et al. 2018; Pérez et al. 2012). For herbivorous reptiles such as *T. graeca* , hindgut nematodes and their associated bacteria might be parasitic while also aiding in the digestion and assimilation of plant matter, which prevents constipation in their hosts. Furthermore, hindgut nematodes regulate the bacterial flora, producing free amino acids for the hosts (Donaldson et al. 2016; Roca 1999), and indicate host‐nematode‐bacteria trophic relationships (Midha et al. 2017; O'Grady et al. 2005; O'Grady and Dearing 2006).

### 2.3 Tortoise Sampling Procedure

Wild tortoises were sampled across 22 different localities (Figure 1), distributed across a mosaic of land‐use practices, including the following: Mediterranean shrublands (original vegetation type) which covered approximately 53% of the sampled area; followed by traditional dryland crops (*secanos*), with 24%; intensified agricultural fields, with 10%; pine plantations, with 7%; and finally, unproductive areas where vegetation cannot grow (e.g., urban zones and infrastructure), with 6%. Mediterranean shrublands and *secanos* are considered as the best available habitats for the maintenance of *T. graeca* wild populations. Sampling localities showed different landscape configuration and composition, as not all land‐use practices were present in the 22 sampling localities concurrently (Appendix 1). Differences in the landscape configuration and composition of transformed Mediterranean shrublands have affected resource distribution and availability, challenging the maintenance of tortoises and other animal populations (Agnoletti and Santoro 2022; Rodríguez‐Caro et al. 2017). Each of the sampling sites covered 1 km^2^, which corresponds to a UTM (Universal Transverse Mercator) cell of 1 × 1 km. The effective search time was at least 7 h and 30 min per site (with three searchers for 2 h and 30 min). Sampling localities ranged from almost undisturbed to highly altered and fragmented landscapes, representing 88.4%–17.4% of the original shrubland cover, respectively. All tortoises sighted were captured and marked by notching the marginal scute. The following individual traits were then recorded: sex, age in years (estimated from the carapace growth ring, following Rodríguez‐Caro et al. 2015), as well as body weight (g), height (mm), plastron width (mm) and carapace length (mm). Thereafter, a growth rate score per individual (mm year‐1, k) was calculated using the von Bertalanffy model (Macale et al. 2009; Zivkov et al. 2007), which assumes age‐related growth rate decline and estimates growth from birth to the time of measurement (Fabens 1965).

### 2.4 Study of Nematode Communities

Individual freshly fecal samples were obtained immediately following the handling of the tortoises or from the cloth bag that contained the tortoises until they defecated. Afterward, individuals were released in their capture sites. After collection, fecal samples were maintained at 4°C for 48 h until processed. Firstly, a saline solution was added to the fecal samples and, throughout microscopic examination, adult nematodes were collected and counted. Secondly, all collected adult nematodes were washed in distilled water and subsequently stored in 70% ethanol (Chávarri et al. 2012). Finally, the nematodes were cleared in Amann's lactophenol solution and then identified to species level according to several taxonomic keys (Bouamer et al. 2001, 2003; Bouamer and Morand 2003, 2006). Two measurements of nematode infestation were considered: prevalence and intensity.

We sampled a total of 116 adults that represented the entire species range of southern Spain (Figure 1). A previous study in the same region showed no effect of tortoise density on nematode infestation (Benítez‐Malvido et al. 2019). Nematode prevalence at young ages is negligible, and therefore, only adults were included in the study: 66 adult females and 50 adult males in total (Chávarri et al. 2012; Benítez‐Malvido et al. 2019). Preliminary analyses showed that female and male individuals were of similar age but differed significantly in all body traits considered (Table 1).

**TABLE 1:** Body traits (mean ± SE) of adult spur‐thighed tortoise populations (*Testudo graeca*) in Mediterranean shrubland landscapes with different land‐use practices in southern Spain.

Tortoise body traits	Females (*N* = 66)	Males (*N* = 50)
Age (years)	14.49 ± 0.43	13.94 ± 0.50
Weight* (g)	656.36 ± 31.20	398.90 ± 21.57
Height* (mm)	77.17 ± 1.57	62.93 ± 1.08
Carapace length* (mm)	139.78 ± 2.61	118.74 ± 1.77
Plastron width* (mm)	69.15 ± 1.16	57.51 ± 0.85

* Significant differences between tortoise sex.

### 2.5 Statistical Analysis

We compared nematode prevalence and intensity between male (*N* = 50) and female (*N* = 66) tortoises through generalized linear models (GLMs) by adjusting data to a negative binomial distribution. Subsequently, to determine the direction of the relationships between different body traits and growth rates (dependent variables) and nematode infestation (independent variable), we compared the linear regression slopes between males and females by employing GLMs with a Gaussian distribution, using all sampled individuals. Nevertheless, a third GLM explored the relationships between land‐use practices and nematode infestation as well as tortoise growth rates between sexes. All analyses used the statistical package R (R Development Core Team 2024). Because nematode infestation across individuals ranged between 0 and 4901, this variable was log(*x* + 1) transformed, while body traits were log(*x*) transformed. The significance was set at *α* = 0.05.

In addition, we performed a principal components' analysis (PCA) using “factoextra” package in R to clearly illustrate sex‐mediated body traits, growth rates and their interactions with nematode infestation. Previously, data were standardized using *Z*‐scores (scale = TRUE, *prcomp()* function) that allow comparing variables measured in different units (i.e., k, mm, g, among others). The biplot was generated through the “fviz_pca_biplot()” in “factoextra,” and “ggplot2” to enhance visual quality. To assess differences between male and female tortoises, a permutational multivariate analysis (PERMANOVA) was performed with the *vegan* package (Oksanen et al. 2025).

### 2.6 Nematode Community Attributes: Species Diversity and Compositional Dissimilarity

To test whether the diversity of endosymbiont nematode species differed between males and females of *T. graeca* tortoises, we employed the effective number of species or Hill series (^*q*^D, Hill 1973; Jost 2006), using only infested individuals. The exponent *q* determines the influence of species abundance on diversity values; ^0^D equals species richness. Furthermore, ^1^D gives a proportional weight to species based on their abundances, which is known as the effective number of equally common species. Conversely, ^2^D gives greater weight to those species that are most abundant in the community, which is known as the effective number of dominant species (Hill 1973; Jost 2006). Evenness is a measure of variation in species relative abundance. When species abundances are perfectly even, the diversity of all orders ^q^D ≥ 0 are identical and equal to species richness. Pielou's *J'* is commonly used as an evenness measure (Pielou 1966) and is expressed as *J'* = log (^1^D)/(logS) = H/(logS), where H denotes Shannon entropy and species richness (S). Therefore, the steepness of the slope reflects the unevenness of species abundances (Chao and Ricotta 2019; Chao et al. 2020). For all values of *q*, species diversity, the evenness profile, and Pielou's measure were computed for a sample coverage value of 99.9% (Ĉmax, Chao et al. 2020). The values of ^*q*^D (±95% CI) and Ĉmax were estimated with the “iNEXT‐4‐steps” package for R (Chao et al. 2020).

To determine the variation of nematode species composition between female and male tortoises, we calculated the compositional dissimilarity based on the Jaccard index (βcc). Compositional dissimilarity (βcc) is broken down into two additive components: replacement (βrepl, species replacement) and richness difference (βrich, loss or gain of species) (Carvalho et al. 2012, 2013; Podani and Schmera 2011). Compositional dissimilarity was calculated with the *betamulti* function in the “BAT” package for R (Cardoso et al. 2015). The variable studied was the average number of hindgut nematode species per host tortoise and sex. The three methods for estimating nematode species diversity were unbiased by host sample size (Chao and Jost 2012; Chao and Ricotta 2019).

### 2.7 Individual‐Based Tortoise‐Nematode Interaction Networks

We incorporated a network approach for investigating the structure of individual‐based tortoise‐nematode networks and their underlying mechanisms between sexes (Dallas and Jordano 2021). For this, we constructed two independent weighted interaction networks, one for each sex. Each sex individual‐based tortoise‐nematode network was constructed through an adjacency matrix *A*, where *a*_*ij*_ = the number of interactions from an individual tortoise *j* by the nematode species *I* or otherwise 0 (i.e., the absence of interaction). In these networks, nematode species and tortoises were depicted as nodes, and their interactions represented by links describing the infestation of tortoise individuals by nematode species. For each sex, the weighed nestedness (*WNODF*) metric evaluated whether or not the nematodes infecting fewer tortoises represented subsets of the tortoises that were infected by a broader number of nematodes (Almeida‐Neto and Ulrich 2011). The core‐periphery measure was evaluated to determine the core nematode groups (i.e., highly connected nodes) and peripheral groups (i.e., poorly connected nodes) according to Dáttilo et al. (2013).

Other network parameters that described their structure were: (i) connectance (the proportion of realized links from the pool of all possible links between the species in the network) and (ii) interaction diversity (*ID*) based on the Shannon diversity index. Higher connectance suggests greater community stability, which reflects a greater spread of interactions among the species in the community. Interaction diversity ranges from 0 (no diversity) to infinity. Higher *ID* is also usually associated with a higher community stability (Blüthgen et al. 2006).

## 3 Results

The majority (68%) of sampled tortoises were infested by nematodes. Regarding sex, 43 out of 66 females and 34 out of 50 males were infested. The mean (±SD) intensity of infestation was 183.01 ± 93.80 for females and 129.66 ± 74.46 for males; however, this difference was not significant (*χ* ^2^ = 124.39, df = 1, *p* = 0.533). The frequency of nematode species varied between 0 and 10 species for either males and females. The abundance of nematodes varied between 0 and 3368 individuals in males and between 0 and 4901 in females. Overall, the relationship between tortoise's body traits, growth rates, and nematode infestation was sex‐biased and influenced by the proportion of unproductive habitat.

### 3.1 Sex, Body Traits, Growth Rates and Nematode Infestation

Infestation predicted changes in body traits and growth rates, revealing sex‐dependent effects. Body weight and carapace length were both significantly influenced by tortoise sex (*F*_1, 103_ = 37.84, *p* = 0.001 and *F*_1, 103_ = 31.71, *p* = 0.001, respectively) only for males. Specifically, males presented a significant negative relationship between infestation intensity, body weight, and carapace length, whereas no such relationship was observed for females (Figure 2a,b, Appendix 2).

**FIGURE 2:**
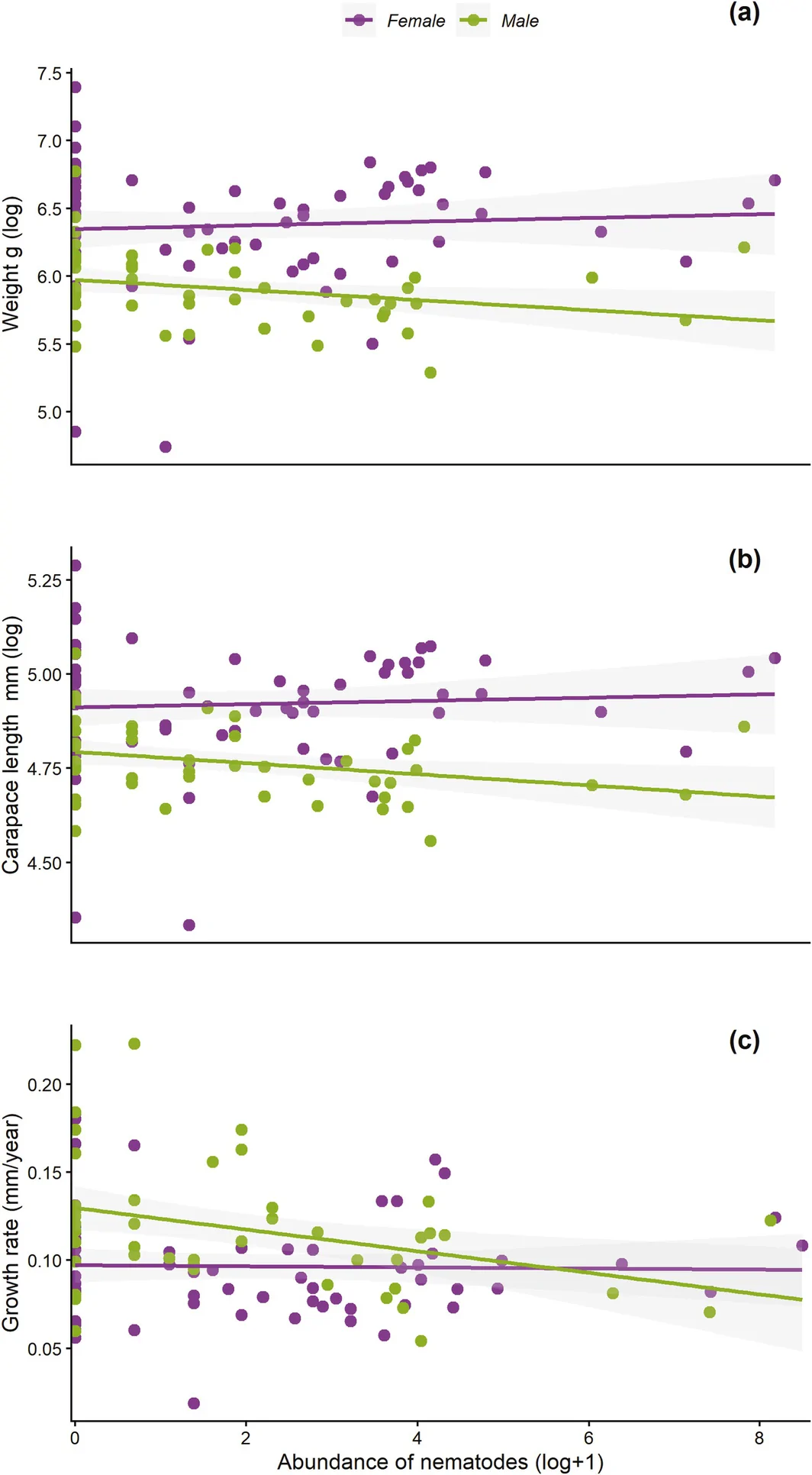
Best‐fit linear regressions between nematode infestation (i.e., abundance of nematodes) and male and female *Testudo graeca* tortoises in southern Spain: (a) weight, (b) carapace length, and (c) growth rates.

Growth rates were affected by nematode infestation intensity depending on tortoise sex (*F*_1, 103_ = 9.247, *p* = 0.003). At low levels of infestation, males grew faster than females, but this pattern reversed as infestation levels increased (Figure 2c). Thus, as infestation increased, males exhibited slower growth while female growth was unaffected. Moreover, growth rates were positively affected by the proportion of unproductive land, regardless of sex (*F*_1, 103_ = 5.23, *p* = 0.024), but males grew significantly faster than females (*F*_1, 103_ = 7.63, *p* = 0.007) (Figure 3a). Finally, infestation intensity decreased as the proportion of the unproductive land increased (*F*_1, 103_ = 10.86, *p* = 0.001), which was also consistent in both sexes (Figure 3b; Appendix 2).

**FIGURE 3:**
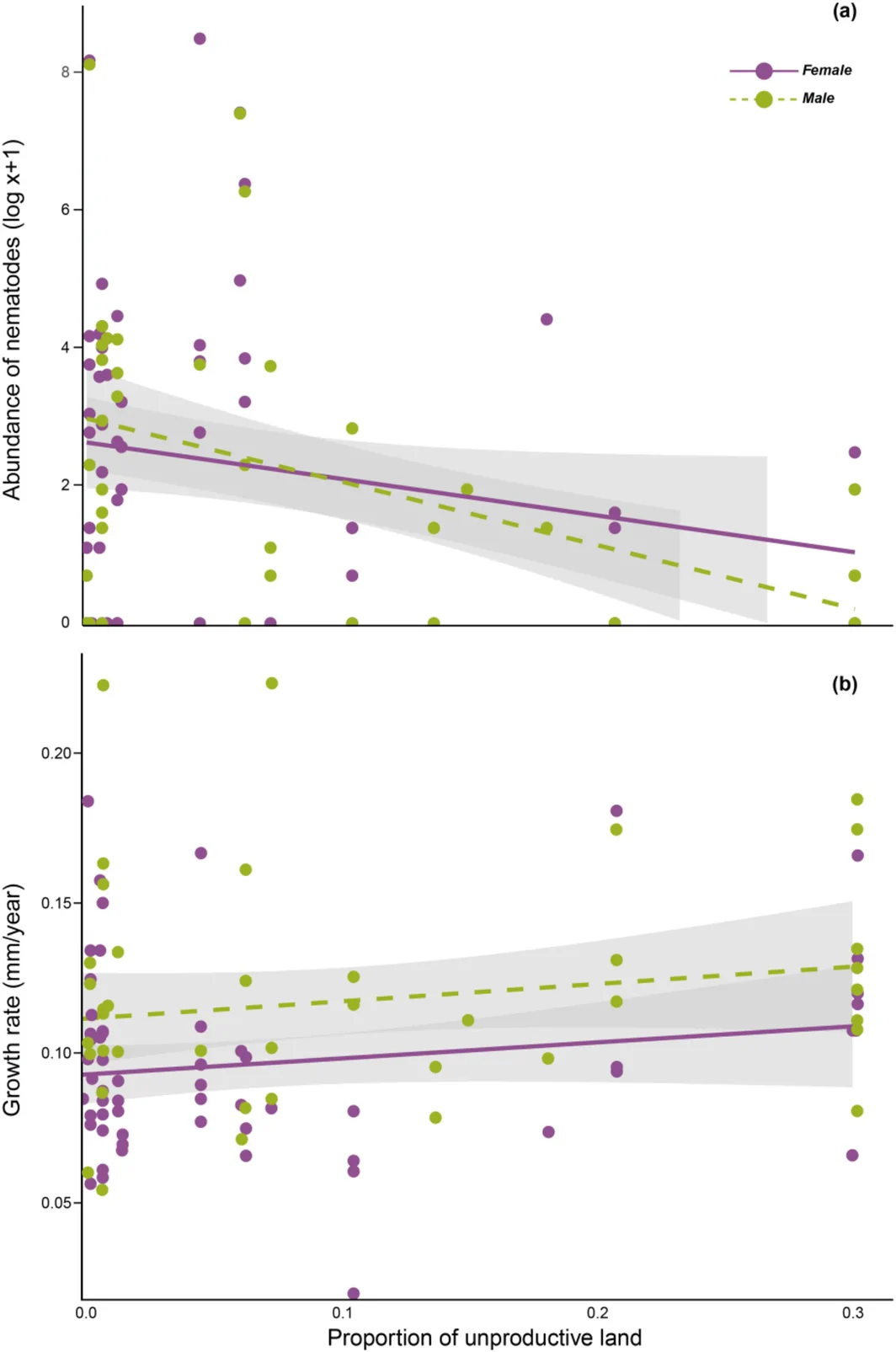
Best‐fit linear regressions between the proportion of unproductive land with (a) nematode infestation (i.e., abundance of nematodes) and (b) tortoise growth rates. Adult female and male tortoises ( *Testudo graeca* ) were present in Mediterranean landscapes of southern Spain with different levels of habitat loss.

The PCA supports our findings. The first two principal components (PCA) explained 78.3% of all data variations (Figure 4), clearly splitting tortoises by sex. The first component (Dim 1) explained 59.4% of the variance, and the biplot showed two clearly distinct groups: females and males. Larger, heavier females and faster growing males were grouped to the left of the biplot. Moreover, the second component (Dim 2) explained 16.9% of the variance, indicating a trade‐off between tortoise growth rates and nematode infestation. Tortoise growth rates and nematode infestation were the variables that showed the longer vectors contributing the most to the observed variation between sexes (Figure 4). The results of the PERMANOVA showed that male and female tortoises differed significantly in their body traits, growth rates and nematode infestation (*F*_1, 102_ = 22.34, *p* < 0.001).

**FIGURE 4:**
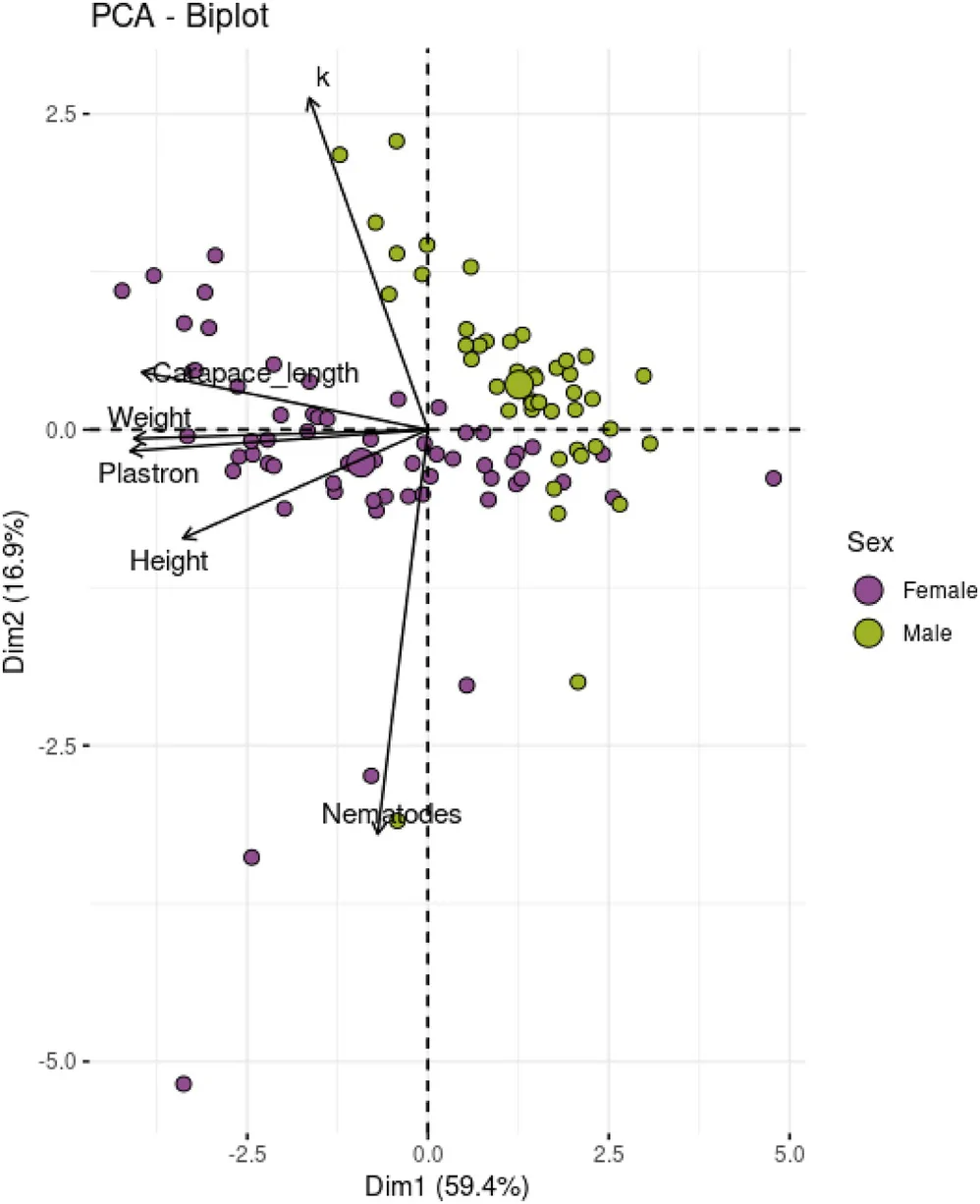
Biplot of principal components´ analysis (PCA) for male and female spur‐thighed tortoises ( *Testudo graeca* ) body traits, growth rates (*k*) and nematode infestation (i.e., species abundance) in southern Spain. Adult female and male tortoises inhabited landscapes sustaining different levels of habitat loss. Larger circles indicate the centroids of the scattered points representing male and female tortoises. “Nematodes” refers to infestation intensity.

### 3.2 Nematode Community Attributes: Species Diversity and Compositional Dissimilarity

A total of 15 adult nematode species (all within the order Oxyurida and the Family Pharyngodonidae) were identified in the feces of wild *T. graeca* populations. Overall, nematode species richness (^0^D), the effective number of equally common species (^1^D), and the dominant species (^2^D) were significantly higher in females than in males (Figure 5, ^q^D ± 95%). Furthermore, Pielou's evenness was slightly higher for females than for males (0.58 vs. 0.53, respectively). All measures consistently showed that evenness among nematode species abundance in females was higher than in males for a standardized 99.9% assemblage fraction (Figure 5). Compositional dissimilarity, however, was low between sexes (βcc = 6.0%; βrepl = 0%; βrich = 6.0%); neither species replacement nor richness differences were detected. One female harbored one unique nematode species (*Thaparia thapari*), whereas males had none.

**FIGURE 5:**
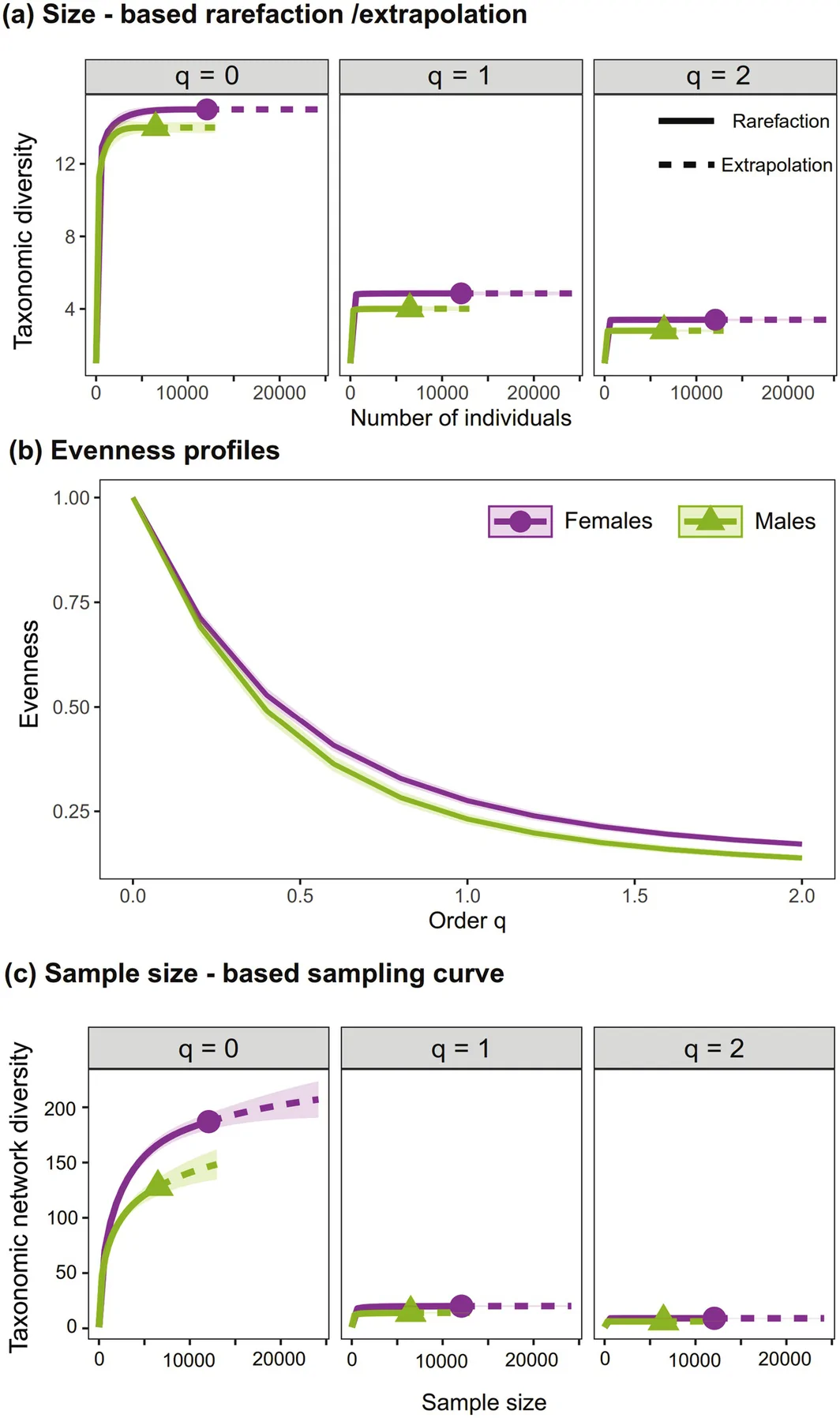
The adult nematode community infesting male and female tortoises ( *Testudo graeca* ) in Mediterranean landscapes of southern Spain. (a) Size‐based rarefaction and extrapolation curves comparing species diversity (^q^D) of nematode communities between sexes. Nematode species richness (^0^D ± 95% CI), the effective number of equally common species (^1^D ± 95% CI), and the effective number of dominant species (^2^D ± 95% CI). (b) Evenness profiles between tortoise's sex (95% CI). (c) Sample‐size‐based rarefaction and extrapolation curves comparing taxonomic network diversity (TND) of individual‐based nematode‐tortoise networks: rare interactions (q = 0), common (q = 1) and dominant (q = 2) interactions.

### 3.3 Tortoise‐Nematode Ecological Networks

We found a significant nested pattern in individual‐based tortoise‐nematode networks in both females and males (Figure 6 and Table 2). Network attributes for both sexes presented similar values of connectance. Nestedness and interaction diversity, however, were greater in females than in males (Table 2). The nematode species *Tachygonetria dentata* and *T. longicollis* were core species for both female and male networks (Figure 6). In female tortoises, the abundance of both core species was similar, whereas *T. longicollis* was more abundant than *T. dentata* in male tortoises. Nematode species turnover as core/periphery components in female and male networks was very low.

**FIGURE 6:**
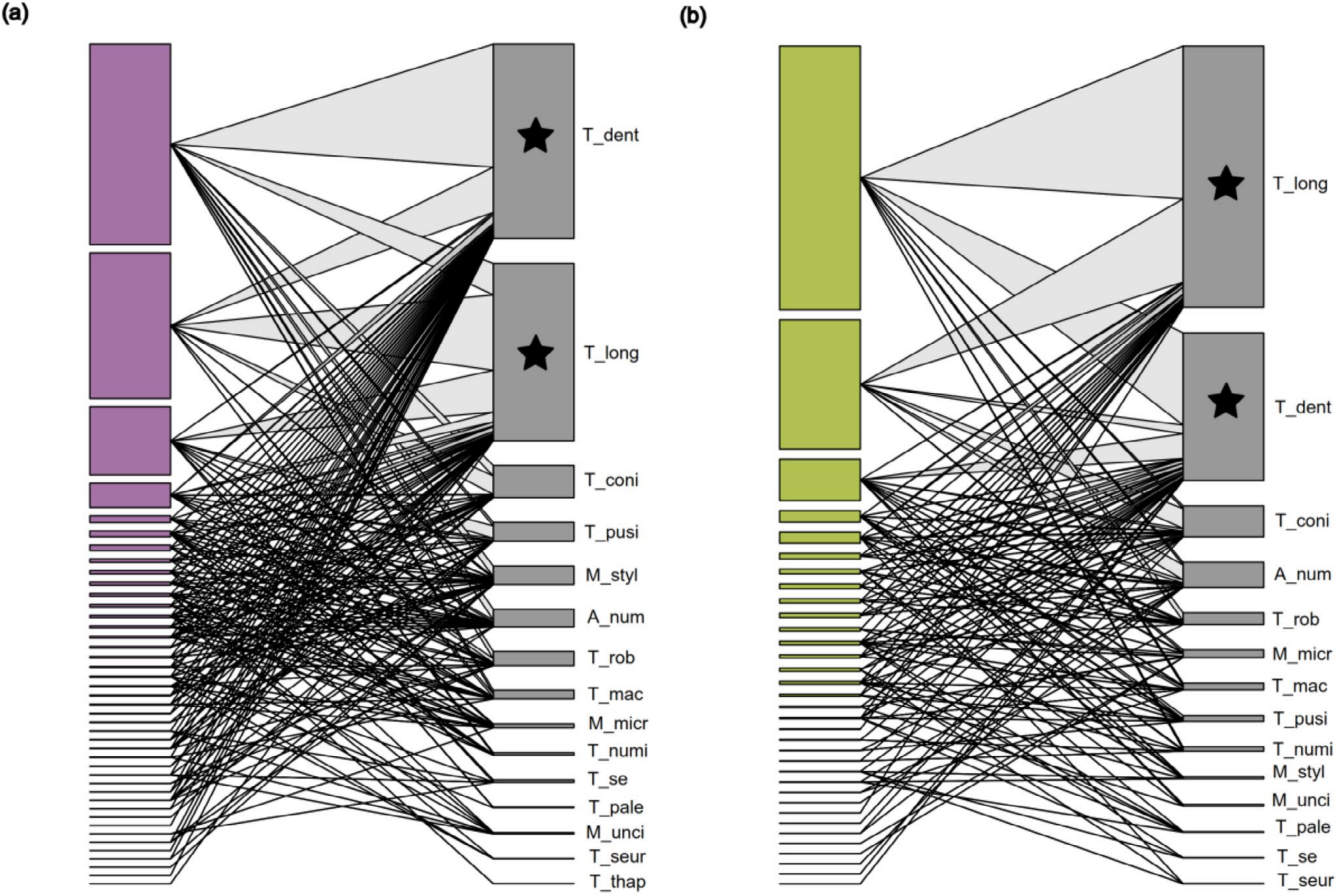
Quantitative bipartite plots of the tortoise‐nematode species network for (a) female tortoises and (b) male tortoises in southern Spain. Bar size represents the number of interactions each nematode species (gray boxes) had with each individual tortoise, whereas line widths (link weight) represent the proportion of nematode individuals involved in such interactions. Nematodes included the following species: *Alaeuris numidica* (*A. num*), *Mehdiella stylosa* (*M. styl*), *M. uncinata* (*M. unci*), *M. microstoma* (*M. micro*), *Tachygonetria longicollis* (*T. long*), *T. dentata* (*T. dent*), *T. conica* (*T. coni*), *T. robusta* (*T. rob*), *T. macrolaimus* (*T. mac*), *T. numidica* (*T. numi*), *T. setosa* (*T. set*), *T. palearticus* (*T. pale*), *T. pusilla* (*T. pusi*), *T. seurati* (*T. seur*), and *Thaparia thapari* (*T. thap*).

**TABLE 2:** Attributes of *Testudo graeca* tortoise‐nematode ecological networks in male and female individuals inhabiting Mediterranean shrublands of southern Spain.

Metrics	Females	Males
No. of tortoises	43	34
Nematode species	15	14
Nestedness (*WNODF*)	**41.06**	**34.50**
Connectance (*C*)	0.29	0.27
Interaction diversity (*ID*)	3.92	3.26

*Note:* Bold values indicate significant nestedness.

## 4 Discussion

Overall, results showed that host responses to nematode infestation are clearly sex‐biased in *T. graeca* , with nematodes exhibiting an antagonistic relationship in males but a commensal one in females (Figures 2, 3, 4). This pattern may result from a complex interplay of sex‐dependent behavioral and ecophysiological factors and the nematode community (Dallas et al. 2018; Dallas and Jordano 2021; Ezenwa and Jolles 2011; Fischhoff et al. 2020; Vayssier‐Taussat et al. 2014). Moreover, we found a landscape effect on both the nematode load and on tortoise growth rates. This suggests that habitat quality is extremely important not only for the tortoises themselves but also for their biotic interactions.

### 4.1 Behavioral and Ecophysiological Factors Affecting Tortoise‐Endosymbiont Interactions

The results showed a negative effect of nematode infestation on body traits and growth rates, in male tortoises only (Figure 2). The interactions between hindgut nematode communities and host ecophysiology are highly complex, especially in the present case where much remains to be learned about the nature of the interaction (commensalistic, mutualistic or antagonistic) and its dependence on behavior and environmental processes. We hypothesized that nematode communities influence growth patterns and size, but an inverse cause‐and‐effect relationship could occur as well: that the nematode community depends on the tortoise's developmental stage and life history. Therefore, future research on the nature and mechanisms of interactions between the host and the nematode community is certainly necessary.

During development and growth, animals confront infection by many different symbiont species that can affect their fitness (Aguirre and Tabor 2008). Shifts in innate or acquired immunity with age sometimes determine age biases in parasitism (Cattadori et al. 2005; Forbes 2020). In *T. graeca* , larger and older individuals bear greater hindgut nematode infestation (Chávarri et al. 2012; Morand and Poulin 1998). In the present study, however, male and female tortoises were similar in age and exposure to nematodes, yet only males were smaller and grow more slowly with higher infestation (Table 1, Figure 2c). This suggests a lower resistance to infection. Transmission routes of hindgut nematodes in *T. graeca* are orofaecal, geophagic and/or sexual (Roca 1999). Coprophagy may help young tortoises develop their microbiota for digesting plant matter successfully as adults (Donaldson et al. 2016; Midha et al. 2017; Rataj et al. 2011). Sexual nematode transmission might be more important for mating adult tortoises, and adult females might ingest soil more frequently than males to support eggshell formation. Additionally, host diet may affect endosymbiont species richness if there is increased food consumption leading to higher nematode ingestion rates (Arneberg 2002; Roca 1999).

### 4.2 Habitat Loss and Tortoise‐Endosymbiont Interactions

Sex‐related differences in habitat preferences shaped by behavioral, physiological, and ecological factors have been documented in wild tortoise populations (Anadón et al. 2012; Bolnick et al. 2003; Dallas and Presley 2014; Forbes 2020; Tompkins et al. 2011). These differences may also influence patterns of nematode transmission. Among the various land‐use types, only unproductive lands significantly affected tortoise‐nematode interactions in both sexes. Both male and female tortoises showed faster growth rates in highly degraded landscapes, despite being exposed to lower nematode loads (Figure 3), as previously found (Benítez‐Malvido et al. 2019). This might be attributed to the more effective body temperature regulation when scrubland vegetation cover is non‐existent (Poulin and George‐Nascimento 2007; Sato et al. 2014). An adequate thermoregulatory behavior has been found to optimize digestion in some reptile species, which affects growth rates (Anguilletta Jr. et al. 2002; Avery et al. 1993). In unproductive lands, tortoises might thus have allocated more resources to growth rather than to defense, thereby bearing a lower nematode load. Furthermore, unproductive lands are mostly devoid of vegetation, litter layer, and exposed soil, where many parasites, including nematodes, may spend part of their life cycles. For another reptile species, the lizard *Psammodromus algirus*, reduced ectoparasite infestation has been reported in a Mediterranean ecosystem after wildfires as compared with unburned areas (Álvarez‐Ruiz et al. 2021).

The distribution of parasites and their hosts is strongly related to the environment (e.g., temperature, land elevation, landscape composition and configuration), to their evolutionary history, and to species ecological traits that drive interaction patterns involving different spatiotemporal processes (Collinge et al. 2005; Forbes 2020; Kramer‐Schadt et al. 2009). The impact of landscape modification on movement and behavioral patterns can be strongly sex‐biased and may influence parasite loads and their richness (Anadón et al. 2012; Fischhoff et al. 2020; Kramer‐Schadt et al. 2009; Nielsen et al. 2020). Probably tortoises move across the mosaic of different land uses common in Mediterranean landscapes and may encounter new suitable habitat patches, which are crucial for their persistence (Anadón et al. 2012; Jiménez‐Franco et al. 2022; Wintle et al. 2019). Contemporary Mediterranean landscapes include several land‐use practices showing a negative relationship between nematode infestation and the proportion of unproductive lands, which apparently represent harsh environmental conditions for both nematodes and tortoises.

### 4.3 The Endosymbiont Nematode Community

Community diversity metrics may improve our understanding of nematode species coexistence, turnover and transmission (Ezenwa and Jolles 2011; Dallas et al. 2018; Hellgren et al. 2004; Runghen et al. 2021). Our findings show that despite the similar nematode infestation all diversity (^q^D) and evenness metrics of the nematode community were greater in female rather than in male tortoises, whereas compositional dissimilarity was very low with negligible nematode species turnover between sexes (Figure 5). Except for *T. thapari*, the same species of nematodes infected both sexes. The species, *T. thapari*, has been previously recorded at low numbers for captive *T. graeca* only, but may infect females as well as males (Chávarri et al. 2012).

The more evenly distributed nematode species in female than male wild tortoises may indicate that female hindguts can sustain a more diverse nematode community and different interaction types between nematodes and their hosts (e.g., mutualistic, antagonistic and commensal ones). In fact, there is evidence that nematode species competition could explain the differential numbers of some of them (e.g., *A. dactyluris*, *M. uncinata* and *T. dentata* ; Johnson and Buller 2011; Midha et al. 2017). Many diseases are the result of interactions within multiple endosymbiont species where the effect of one pathogen species (e.g., nematode) could be moderated by another one (Ezenwa and Jolles 2011; Midha et al. 2017; Vayssier‐Taussat et al. 2014). Furthermore, competition between nematode species could be driven by environmental conditions under which the host lives. Therefore, to understand the ecology of diseases (e.g., the pathobiome), integrating the interaction among endosymbionts, hosts, and the environment is necessary (Dallas and Presley 2014; Ezenwa and Jolles 2011; Johnson and Buller 2011).

A differential response to a similar pathogen infestation because of sex‐specific behavior and physiology has been described as influencing parasite species richness and persistence (Declerck et al. 2014; Ezenwa and Jolles 2011; Nielsen et al. 2020). Female resource allocation strategies focus on locating resource‐rich sites for feeding and oviposition, whereas males prioritize mate searching. Additionally, sex‐biased spatial distribution of hosts may affect parasite communities (Declerck et al. 2014; Gosnell et al. 2009; Nielsen et al. 2020).

### 4.4 Tortoise‐Nematode Interaction Networks

The nematode community infesting *T. graeca* populations was not randomly assembled but nested, revealing a structured pattern of interactions for both sexes. Tortoise‐nematode network properties appear to be maintained in both sexes despite contrasting differences in body conditions and growth rates (table 2; Dallas and Jordano 2021). The greater nested pattern in females implies that nematodes could be more easily transmitted through the entire network: nodes with few links (specialist nematodes) usually associate with generalist partners, such as with hosts that bear high nematode richness. This favors coinfections across host tortoise populations (Dallas and Jordano 2021; Hudson et al. 2006). The similar number of nematode species found in both sexes suggests that *T. graeca* maintains a stable nematode community regardless of sex (Figure 6). The lowest diversity of interactions found in the male networks suggests that they are less stable than female networks. The nematodes *T. dentata* and *T. longicollis* were core species for both sexes and supported the nested pattern in the networks. These species could be playing a mutualistic role as well by contributing nitrogenous excretions that enhance the digestive system of tortoises (Roca 1999).

## 5 Conclusions

Our findings contribute to understanding the behavioral and ecophysiological aspects of sex in defining the endosymbiont community in the spur‐thighed tortoise and linking tortoise‐nematode interactions. The influence of nematode infestation on *T. graeca* appears to be highly variable and context‐dependent. Negative effects of parasites on individual fitness should be most concerning for vulnerable and endangered species such as chelonians worldwide (Fischhoff et al. 2020; Lovich et al. 2018; Stanford et al. 2020). Furthermore, parasites could potentially affect the structure and stability properties of wildlife communities, which could be further exacerbated by habitat degradation (Arneberg 2002; Dallas et al. 2018; Dallas and Presley 2014). By examining wild tortoises, we could determine that infestation susceptibility was sex‐biased, with males exhibiting a parasitic relationship as their growth rates declined with increasing parasite load. Females, however, showed a commensal interaction since their growth rates remained unaffected. We also found a significant effect of habitat loss, as unproductive lands posed harsh environmental conditions for both nematodes and tortoises of both sexes. Sex differences in parasite infestation and its implications for animal fitness have probably evolved just as sex differences in morphology and behavior have, which comes from natural selection acting differently on males and females (Nielsen et al. 2020). We conclude that considering tortoise sex is vital when applying management tools for conservation, as different sexes respond differently to nematode infestation, as well as habitat quality involving land uses that are essential for sustaining species survival and their interactions.

## Author Contributions

**Julieta Benítez‐Malvido:** conceptualization (lead), data curation (lead), formal analysis (lead), funding acquisition (supporting), investigation (supporting), methodology (supporting), project administration (supporting), resources (supporting), software (lead), supervision (supporting), validation (lead), visualization (lead), writing – original draft (lead), writing – review and editing (lead). **Eva Graciá:** conceptualization (equal), data curation (lead), formal analysis (supporting), funding acquisition (lead), investigation (lead), methodology (lead), project administration (lead), resources (lead), software (supporting), supervision (lead), validation (lead), visualization (lead), writing – original draft (supporting), writing – review and editing (equal). **Andrés Giménez:** conceptualization (equal), data curation (equal), formal analysis (supporting), funding acquisition (lead), investigation (lead), methodology (lead), project administration (lead), resources (lead), software (supporting), supervision (equal), validation (equal), visualization (equal), writing – original draft (supporting), writing – review and editing (equal). **Iraís Ávila‐Eulogio:** conceptualization (supporting), data curation (equal), formal analysis (lead), funding acquisition (supporting), investigation (supporting), methodology (supporting), project administration (supporting), resources (supporting), software (equal), supervision (supporting), validation (equal), visualization (equal), writing – original draft (supporting), writing – review and editing (supporting). **Diana María Méndez:** conceptualization (supporting), data curation (lead), formal analysis (lead), funding acquisition (supporting), investigation (supporting), methodology (supporting), project administration (supporting), resources (supporting), software (lead), supervision (supporting), validation (supporting), visualization (supporting), writing – original draft (supporting), writing – review and editing (supporting). **Roberto Carlos Rodríguez‐Caro:** conceptualization (supporting), data curation (lead), formal analysis (lead), funding acquisition (supporting), investigation (lead), methodology (lead), project administration (supporting), resources (supporting), software (supporting), supervision (supporting), validation (supporting), visualization (supporting), writing – original draft (supporting), writing – review and editing (equal). **Rocío Ruiz‐De Ybáñez:** conceptualization (supporting), data curation (lead), formal analysis (supporting), funding acquisition (supporting), investigation (supporting), methodology (lead), project administration (supporting), resources (supporting), software (supporting), supervision (supporting), validation (supporting), visualization (supporting), writing – original draft (supporting), writing – review and editing (supporting). **Anna Traveset:** conceptualization (supporting), data curation (supporting), formal analysis (supporting), funding acquisition (supporting), investigation (supporting), methodology (supporting), project administration (supporting), resources (supporting), software (supporting), supervision (supporting), validation (supporting), visualization (supporting), writing – original draft (supporting), writing – review and editing (equal).

## Funding

Financial support was awarded by project TED2021‐130381B‐I00, granted by the Ministerio de Ciencia, Innovación y Universidades (MICIU/AEI/10.13039/501100011033), with the support of the European Union “NextGenerationEU/PRTR”.

## Conflicts of Interest

The authors declare no conflicts of interest.

## Data Availability

Data generated or analyzed during this study are available in Dryad Dataset Reviewer sharinglink: https://doi.org/10.5061/dryad.d7wm37qhc.

## Appendix 1

The proportion of different land‐use practices present at 22 localities in the semiarid coastal mountains between the Almeria and Murcia provinces, Southern Spain. Individuals of *Testudo graeca* wild tortoises were sampled across a mosaic of land‐use practices. Landscape configuration and composition of transformed Mediterranean shrublands have affected resource distribution and availability, challenging the maintenance of tortoises.

**Table:**

Site number	Name	Pine plantation	Mediterranean shrubland	Un‐productive land	Secano	Intensive agriculture
1	Adanes	0.513	0.473	0.000	0.013	0.000
2	Alboluncas	0.000	0.370	0.301	0.225	0.104
3	Bas norte	0.000	0.508	0.060	0.367	0.065
4	Bas sur	0.000	0.327	0.062	0.431	0.180
5	Chinas	0.000	0.673	0.180	0.147	0.000
6	Cortijo Aire	0.219	0.688	0.008	0.002	0.084
7	Cortijo de Romero	0.000	0.323	0.005	0.597	0.075
8	Cortijo del Tio Rey	0.000	0.285	0.207	0.447	0.061
9	Cuesta del Grajo	0.030	0.884	0.000	0.085	0.000
10	Galera	0.000	0.781	0.006	0.213	0.000
11	La Hoya	0.000	0.244	0.072	0.160	0.523
12	Lentiscar	0.547	0.453	0.000	0.000	0.000
13	Los Porceles	0.005	0.291	0.002	0.639	0.062
14	Marinica	0.000	0.968	0.014	0.019	0.000
15	Nuevo Misiripalme	0.000	0.373	0.012	0.589	0.026
16	Palas	0.000	0.521	0.044	0.046	0.388
17	Pane	0.005	0.174	0.104	0.491	0.225
18	Sierrecica	0.000	0.686	0.136	0.067	0.111
19	Sotomayor	0.124	0.873	0.003	0.000	0.000
20	Tova	0.088	0.683	0.012	0.215	0.003
21	Vilerda	0.004	0.252	0.149	0.485	0.111
22	Villaltas	0.000	0.789	0.001	0.091	0.119

## Appendix 2

Generalized linear models showing the effect of nematode infestation and tortoise sex on body traits and growth rates, including: (a) body weight; (b) carapace length; and (c) growth rates. Furthermore, the effect of the proportion of unproductive land on (d) nematode infestation, and (e) growth rates by tortoise sex, was also analyzed.

(a) Effect of nematode infestation on body weight in male and female tortoises.

**Table:**

Factor	df	*F*	*p*
Abundance of nematodes	1	0.13	0.71
Tortoise sex	1	37.84	0.001
Abundance of nematodes × Tortoise sex	1	3.98	0.17
Error	100		
Total	103		

(b) Effect of nematode infestation on carapace length in male and female tortoises.

**Table:**

Factor	df	*F*	*p*
Abundance of nematodes	1	0.26	0.60
Tortoise sex	1	31.71	0.001
Abundance of nematodes × Tortoise sex	1	2.01	0.15
Error	100		
Total	103		

(c) Effect of nematode infestation on growth rates in male and female tortoises.

**Table:**

Factor	df	*F*	*p*
Abundance of nematodes	1	3.6374	0.0593
Tortoise Sex	1	9.2478	0.0030
Abundance of nematodes × Tortoise sex	1	3.3898	0.0685
Error	100		
Total	103		

(d) Impact of the proportion of unproductive land on nematode abundance in male and female tortoises.

**Table:**

Factor	df	*F*	*p*
Proportion of unproductive land	1	15.4451	0.0001
Tortoise sex	1	0.0001	0.9957
Proportion of unproductive land × Tortoise sex	1	1.0843	0.3002
Error	100		
Total	103		

(e) Impact of the proportion of unproductive land on growth rates in male and female tortoises.

**Table:**

Factor	df	*F*	*p*
Proportion of unproductive land	1	5.23	0.024
Tortoise Sex	1	7.63	0.007
Proportion of unproductive land × Tortoise Sex	1	0.0044	0.9473
Error	100		
Total	103		
